# Long-run causal impact of the 2011 Economic Adjustment Programme on Portuguese population health

**DOI:** 10.1371/journal.pone.0324756

**Published:** 2025-05-30

**Authors:** Aida Isabel Tavares, Noureddine Si Abdallah

**Affiliations:** 1 CEISUC – Centre for Health Studies and Research, University of Coimbra, Portugal; CiBB - Centre for Innovative Biomedicine and Biotechnology, University of Coimbra, Portugal; ISEG, UL - Lisbon School of Economics and Management, University of Lisbon, Portugal,; 2 Department of Applied Mathematics, University of Alicante, Spain; Pusan National University College of Economics and International Trade, KOREA, REPUBLIC OF

## Abstract

In 2011, Portugal signed a bailout programme to be implemented over a 3-year period, from 2011 to mid-2014. This programme included a set of measures to control and reduce the public sector, which included interventions in areas such as public spending and financing, the labour market, education, and health, among other structural budget measures. The aim of this research is to determine the long-run causal impact of implementing this Economic Adjustment Programme on several population health outcomes. Data was collected for the period 1990–2019 for Portugal. Health outcome indicators account for DALY and HLY, among other general health indicators, but also for some specific mortality rates like those for stroke and cancer. Control variables include percentage of population older than 65, percentage of people with a university degree, CO2 emissions for Portugal, and also health outcomes data for Denmark, Netherlands, Norway and Sweden. The analytical method used is based on a Bayesian structural time series model, which builds a contrafactual scenario representing the absence of the Economic Adjustment Programme for comparison with observed data. The most significant result is the negative long-run causal impact arising from the implementation of the Economic Adjustment Programme in 2011 in Portugal; in other words, health improvements would have been better and faster had the bailout programme not been implemented, despite some level of uncertainty regarding the results. Findings reinforce the idea for the implementation of social and health policies that complement IMF bailout programmes to mitigate negative impacts on population health in the long-run.

## Introduction

### Contextual background

The European sovereign debt crisis was a financial turmoil in the EU which began in 2009 and lasted up to the mid-to-late 2010s. During this period, some Eurozone countries, including Portugal, struggled to repay or refinance their government debts. Some countries ended up requesting external assistance from the European Central Bank (ECB) and the International Monetary Fund (IMF) to support their over-indebted banks. The global financial crisis of 2008 exacerbated the weaknesses of the Portuguese economy, revealing its structural vulnerabilities and exposing pre-existing internal and external imbalances.

The Economic Adjustment Programme (EAP) was to be implemented during a 3-year period, from 2011 to mid-2014. It accounted for a total financing package of €78 billion, €52 billion of which was provided by the EU and about €26 billion by the IMF [1,2]. The general goals of this Programme were to push fiscal indicators onto a sustainable footing, to stabilise the financial sector, and to perform profound structural reforms to support a systematic correction of imbalances both internal and external in order to maximize potential growth [1,3]. Specifically, EAP focused on three pillars: fiscal consolidation through deficit reduction and tighter control over public-private-partnerships and state-owned enterprises; financial sector stabilization via bank deleveraging, recapitalization, and enhanced supervision; and structural reforms targeting labour, justice, and key sectors to boost growth and competitiveness [1].

The bailout programme contained a number of measures to control and shrink the public sector, and these included interventions in the areas of public spending and financing, regulation and supervision of the financial sector, the labour market, education, health, housing market, public contracts, and procurement, among other structural budget measures [4–7]. For instance, as stated in the *Portugal - Memorandum of Understanding on Specific Economic Policy Conditionality (MoU)* [4], the measures included: i) fiscal policy actions generally resulting in increased taxes and revenue alongside reduced public expenditure; ii) financial sector regulation and supervision measures aimed at preserving financial sector stability, maintaining liquidity, supporting a balanced and orderly deleveraging process in the banking sector, and strengthening banking oversight; iii) fiscal-structural reforms intended to improve the efficiency of public administration; iv) labour market and pension reforms, including revisions to unemployment benefits, employment protection legislation, working time arrangements, and wage-setting mechanisms, which led to wage and pension reductions, limited access to income support, and extended working hours; v) revised conditions in the markets for goods and services—namely energy, telecommunications, transport, and postal services—which resulted in higher prices for gas, electricity, and telecommunications; and finally, vi) housing market reforms concerning the rental sector, administrative procedures, and property taxation, which led to increased rents and higher property taxes.

**Graph 1:**
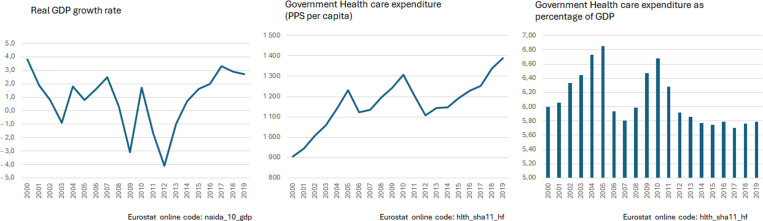
Macroeconomic indicators for Portugal, 2000–2019. The first graph refers to the Real GCP growth rate; the second graph refers to the Government Health care expenditure in PPS per capita; the third graph refers to the Government Health care expenditure as a percentage of the GDP.

The National Health Service (NHS) received special attention resulting from the (public) Beveridge-type characteristics. The measures predicted in the Programme aimed at more than just short-run expenditure savings, they also set out to build mechanisms for future control of health care expenditure in the public sector [8]. These measures, designed to reform the health care sector, were implemented in areas such as funding, pharmaceuticals and prescriptions, primary care services, and hospital services [8–11]. These conditionalities and measures included higher NHS co-payments, stricter exemption criteria, reduced pharmaceutical spending through the promotion of generics and mandatory e-prescriptions, strengthened primary care to reduce specialist consultations, hospital cost reductions via service reorganization and cuts in overtime, and efficiency-enhancing measures such as the implementation of electronic medical records and reduced patient transport costs. The objective of the Programme’s health sector measures was to achieve savings of €550 million in 2012 and €375 million in 2013. In fact, already in 2011, there was a sharp decrease in health expenditures, both as a percentage of GDP and per capita, as illustrated in Graph 1, indicating an abrupt implementation of health system reforms in parallel with the contraction of real GDP (Graph 1).

The Economic Adjustment Programme agreed with the European Commission (EC), the European Central Bank (ECB), and the International Monetary Fund (IMF) in May 2011 implemented a contractionary and pro-cyclical fiscal policy along with a highly restrictive income policy, leading to a sharp decline in domestic demand and macroeconomic deterioration [9,12]. This also caused a reversal of external financing in the Portuguese economy, a situation that was only partially alleviated by the economic and financial assistance programme. The EAP led to a severe recession and deterioration of living conditions [5–7,13] as shown by a drop of 6.8% in GDP at constant 2011 prices, a decrease in GDP real growth and family income, a massive job loss for about 469,000 employees, and a significant rise in the unemployment rate. Youth unemployment peaked at 38.1%, emigration rose significantly, and the risk of child poverty increased by 16.5%. Additionally, the public debt-to-GDP ratio surged from 100.2% in 2010 to 131.4% in 2013.

### Literature overview

The application of IMF structural adjustment programmes in financially constrained countries (such as the EAP implemented in some European countries) required certain conditionalities to promote policy and institutional reforms and achieve short-term economic goals. These conditionalities, which aimed to ensure debt repayment, had an impact on health outcomes due to fiscal austerity and the weakening of health and social systems [14–17]. Three basic pathways can be identified that explain the link between the implementation of these programmes and population health [18]: i) direct impact on healthcare infrastructure (including quality of provision and extent of coverage); ii) direct effect through social determinants of health such as unemployment, education, and poverty; iii) indirect impact via non-health-related policies (Health in All Policies).

These pathways operate across the health determinants as modeled by Dahlgren and Whitehead [19,20], as represented in Picture A1 in the appendix. According to this ecological model centred on the individual, people are surrounded by multiple layers of health influences, including personal lifestyle choices, community factors, living and working environments, and broader social conditions. The initial layer relates to individual behaviour and lifestyle choices that impact health, such as the decision to smoke. These behaviours are shaped by social relationships and community norms. The second layer involves social and community influences, which can offer support during hardship but may also be unsupportive or harmful. The third layer encompasses structural determinants like housing quality, work environments, access to services, and the availability of essential infrastructure.

Accordingly (see Picture A1 in the appendix), there is a direct impact arising from adjustments in the healthcare sector and another direct impact arising from changes in social determinants such as education, work environment, living and working conditions, unemployment, and housing. While the direct impact on the health sector may have resulted in a higher level of unmet healthcare needs and delayed care [10], the impact from other social conditions led to lower disposable income and longer working hours. Finally, the third pathway linking the EAP and population health is the one mediated by policies not directly related to health. This may be best described as the Health in All Policies [21] pathway. It captures the perspective that integrates health considerations into policy making across all sectors to improve population health, including financial sector regulation [22], fiscal policy [23], and utilities market regulation, as established in the EAP [24].

Studies have shown that IMF conditionalities arising from structural adjustment programmes negatively impact population health spending [25] and general health [16]. While most studies are focused on developing countries, developed countries are less analysed as they are likely to face sovereign debt crises, as happened in 2011 in Europe. Both Portugal and Greece have severely experienced this crisis, and both countries have experienced negative effects on health outcomes [10,26]; such effects included increased mortality rates from respiratory diseases, the incidence of infectious diseases, suicide, and road traffic accidents. The worsening of health care [10,11] in these countries was observable by increased waiting times, fewer screenings, and an increase in unmet medical needs.

Despite the clear association between structural adjustment programmes and worse health outcomes, very few empirical analyses have established a causal impact [14–16]. At the most, what is shown is some association or correlation between statistical numbers. In fact, there are two studies by the same authors [14,15] that show the causal harmful effect of IMF programmes on population health. The two studies are a panel data analysis for 187 countries, covering the period 1990–2017; one study focuses on infectious disease mortality, and the other on all causes of mortality rates and the DALY indicator (disability adjusted life years). Both studies use instrumental variable analysis to obtain and proof the existence of a causal relationship, especially for developing countries.

However, this time-series and instrumental variable analysis is susceptible to some limitations related to the choice of the instrument, non-stationarity of time series and autocorrelation, as pointed in classical econometrics [27]. In addition, cross-countries comparisons which differ in institutions and policies may also affect the validity of the instruments [28]. Moreover, the results show a trend which applies mostly to developing countries and not to developed countries. The research presented here is focused on a single developed country, Portugal, and uses a time-series Bayesian approach which, to the best of our knowledge, is the first study in this strand of the literature. The time-series Bayesian approach has been used in several instances to study causal effects in health economics and health sciences [29–34]. In fact, the potential of the analysis has been widely used in the general field of economics [35–39]. The Bayesian approach used in this analysis has the advantage of including local trends, seasonal components, and regression terms with control covariates, which are flexible enough to handle many forms of non-stationarity. Moreover, Bayesian statistical analysis incorporates prior information into the analysis and continuous update, and results interpretation may be more intuitive than the Classical econometrics approach, despite the subjective interpretation [40,41].

### The gap in literature

The overall idea rising from the literature suggests that IMF bailout programmes are detrimental to health outcomes. The expected impact of IMF interventions on health systems includes a reduction in public sector spending which can affect the quality of health care and the social determinants of health, leading to negative health outcomes in the short-run due to worsening in the equity and accessibility of healthcare services. In addition, empirical analysis indicates that the debt burden from IMF loans can constrain government health and social spending in the long run, as countries have to allocate more resources to servicing the debt rather than investing in social and health capital [14–17]. However, previous research usually establishes correlations, and it seldom establishes a well-funded causal relationship where worse health outcomes arise due to IMF programmes in specific countries. Our investigation uses recent causal analysis methods, particularly a Bayesian structural time series model, to establish the causal long-run effect and estimate the health burden caused by IMF bailout programmes.

The aim of this study is to test the potential long-term impact of the 2011 IMF bailout programme in Portugal on population health, measured by different indicators. The hypothesis is the existence of a causal effect between the implementation of IMF programme and health outcome indicators, such as DALY (disability adjusted life years) and HLY (healthy life expectancy at birth), which are of major importance among population health indicators. The results allow a comparison between the original time series of each indicator and the estimated time series adjusted by Bayesian algorithm after the implementation of the IMF programme.

We collected data for Portugal for the period 1990–2019 and applied an algorithm based on the Bayesian structural time series model developed by Brodersen and his team [40] to a set of health outcome indicators.

The main finding of this work is the detrimental long-run causal effect of the IMF bailout programme on population health. To the best of our knowledge, this is the first study using causal analysis applied to several health indicators for a single country experiencing an IMF bailout programme.

## Materials and methods

### Outcome variables and response series

The set of eleven outcome variables used in this analysis are described in Table 1.

**Table 1:** Population health variables description.

Variable	Abbreviation	Description	Source
*Population health indicators*			
Disability-Adjusted Life Years	DALY	Disability-adjusted life years per 100,000 people from all causes (rate) measures the total burden of disease – both from years of life lost due to premature death and years lived with a disability.	OWD
Healthy life expectancy at birth	HLY	Healthy life expectancy measures the estimated average number of years lived free from disability or disease burden. This is based on period life expectancy, after adjusting for the number of years lived in less than ‘full health’ due to disease and/or injury, if the rates of all-cause mortality and all-cause disability in a specified year of interest would remain constant into the future.	OWD
Life expectancy at birth	LEB	The period of life expectancy at birth, in a given year (number of years).	OWD
Life expectancy at age 65	LE65	The total period of life expectancy at age 65, in a given year (number of years).	OWD
*Specific mortality indicators*			
Infant mortality rate	IMR	The estimated number of deaths of children aged under one year, per 100 live births (rate).	OWD
Cardiovascular diseases deaths	CVD_D	Age-standardized deaths that are from cardiovascular diseases per 100,000 people [Cardiovascular diseases have the following ICD 10 codes: I00-I99).	OWD
Lower respiratory infections deaths	Respiratory_D	The estimated number of age-standardized deaths from lower respiratory infections, per 100,000 people, age-standardized (Rate) (Pneumonia accounts for the majority of lower respiratory infections.)	IHME-GBD
Stroke deaths	Stroke_D	Age-standardized deaths from stroke, age-standardized per 100,000 people (rate)	OWD
Cancer deaths	Cancer_D	Age-standardized deaths from malignant neoplasms per 100,000 people (rate)	OWD
HIV/AIDS deaths	HIV_D	Age-standardized estimated deaths from HIV/AIDS per 100,000 people (rate)	IHME-GBD
Tuberculosis deaths	TB_D	Age-standardized estimated deaths from tuberculosis per 100,000 people (rate)	IHME-GBD

Note: OWD – Our World in Data[42]; IHME-GBD Institute for Health Metrics and Evaluation, Global Burden of Disease [43].

The health outcome variables analysed include general population health indicators – DALY (disability adjusted life years), HLY (healthy life expectancy), LEB (life expectancy at birth), LE65 (life expectancy at 65) and IMR (infant mortality rate) and a set of specific mortality indicators were collected according to data availability: mortality rate from cardiovascular diseases, stroke, cancer, respiratory diseases, tuberculosis, and HIV/AIDS for Portugal for the period 1990–2019. The period begins in 1990 due to data availability. The last considered year is set at 2019 to prevent any confounding effects from the COVID-19 pandemic, from 2020 on.

### Control variables and series

The control variables for Portugal are the percentage of population older than 65, the percentage of population completing tertiary level of education, and the CO2 emissions, as described in Table 2. On the one hand, these variables are well-known determinants of population health [19].

**Table 2:** Control variables description.

Variable	Abbreviation	Description	Source
% population +65	POP65+	Percentage of population aged 65 years and more.	Eurostat
% population tertiary level of education	Education	Percentage of population with tertiary education.	INE
CO2 emissions	CO2	CO2 emissions of fossil origin (tonnes per inhabitant).	INE

Note: Eurostat is the European Union Statistical Office[50]; INE - Statistics Portugal, the Portuguese national statistical office [51].

On the other hand, these three variables were not affected nor are they correlated with the implemented EAP in 2011 because they are shaped by structural, demographic, and historical factors that evolve over decades, making them less responsive to economic crisis. Population aging is a long-term demographic trend, education patterns reflect past investment in education and long-term societal trends, and CO2 emissions are linked to industrial activity and long-term energy, transportation and regulation patterns [44–46].

The control variables include the series for the same eleven health outcome indicators for Sweden, Denmark, Norway, and Netherlands, for the same period 1990–2019 (which totals 44 control time series). The reason to consider these countries is that they were not affected directly by the sovereign debt crisis happening in Southern European countries. In these countries, a combination of monetary independence, prudent fiscal management, robust economic structures, and effective crisis response strategies enabled them to avoid the direct impacts of the sovereign debt crisis that affected southern European countries. [47–49]. Additionally, Sweden and Denmark chose not to adopt the euro, maintaining their own currencies allowed them to implement tailored fiscal and monetary policies, insulating their economies from euro-zone-specific issues [47–49]. Finally, the case of Sweden is worth noting because it is a benchmark for Portugal as it has an identical population size and the health system is financed by taxes, as is the Portuguese health system.

### Intervention considered

The intervention considered is the implementation of the EAP in 2011. Thus, the pre-intervention period is defined as 1990–2011, and the post-intervention period as 2012–2019, when the effects of the EAP are expected to emerge. It is worth mentioning that the post-intervention period is reasonably stable in terms of external shocks, such as medical advances [52], which suggests that the effects observed in this period are comparable to those captured in the pre-intervention period. Accordingly, deviations between observed outcomes and the counterfactual can be attributed to the EAP. Finally, any possible effects from the global financial crisis of 2007 are incorporated into the observed data during the pre-intervention period.

### Statistical analysis - Causal impact analysis

Causal Impact is a Google-developed algorithm designed to form a Bayesian structural time series model using several control groups to predict baseline values for the post-intervention period. This model aims to estimate the counterfactual, meaning it predicts how the response metric would have changed after the intervention if the intervention had not taken place, and it was developed by Kay Brodersen and a team from Google [40]. The implementation of this analysis is performed by an R package called CausalImpact.

This analysis requires a time series in which the event occurred, a set of time series in which the event did not occur, representing the control variables and the date the event occurred. The Causal Impact approach builds the predicted time series using a Bayesian structural time series model based on the relationship between the time series in which the event occurred and the control variables.

The Bayesian structural time series model considered is described as follows:


$$\begin{array}{c} y_{t}={Z_{t}}^{T}\alpha_{t}+\varepsilon_{t},\;\varepsilon_{t}N(0,{\sigma_{t}}^{2})-observationequation, \end{array}$$

$$\begin{array}{c} \alpha_{t+1}=T_{t}\alpha_{t}+R_{t}\eta_{t},\;\eta_{t}N\left(0,Q_{t}\right)-stateequation, \end{array}$$

where ε_t_ and η_t_ are independent of all other unknowns.

The first equation is the observation equation; it links the observed data y_t_ to a latent d-dimensional state vector α_t_. The second equation is the state equation; it governs the evolution of the state vector α_t_ through time. In the present study, y_t_ is a scalar observation, Z_t_ is a d-dimensional output vector, T_t_ is a d × d transition matrix, R_t_ is a d × q control matrix, ε_t_ is a scalar observation error with noise variance σ_t_, and η_t_ is a q-dimensional system error with a q × q state-diffusion matrix Q_t_, where q ≤ d.

The Causal Impact approach essentially uses two Bayesian techniques:

i) First, the spike-and-slab regression is a Bayesian technique that deals with control variables. It helps to find the values of Z_t_ and simultaneously reveals the ‘importance’ of each time series in the control variables relative to the target time series. The ‘spike’ is the probability that a given regression coefficient is zero and the ‘slab’ is the past distribution of coefficient values. Based on this probability and distribution, the spike-and-slab regression ranks the importance of the time series in which the event did not occur relative to the target series. That is, after the selection of these time series, algorithm used studies and categorizes them according to the importance of each time series, and hence the package determines the degree of their inclusion in the construction of the model. That is, synthetic counterfactuals are created by a regression component that selects relevant covariates using a spike-and-slab prior to the regression coefficients.
ii) Second, Bayesian model averaging is used to calculate the other model parameters by combining the samples obtained from repeated Markov Chain Monte Carlo (MCMC) draws. This computation generates the synthetic counterfactuals for the post-treatment period based on both the post-period control series and the pre-period response and control series. The intervention effect is then identified as the difference between the actual and counterfactual time series during the post-period.

Finally, it should be noted that the CausalImpact algorithm does not require the treated or control time series to be stationary, as it relies on Bayesian structural time series models. These models incorporate local trends, seasonal components, and regression terms with control covariates, offering sufficient flexibility to accommodate various forms of non-stationarity, including random walks.

### Assumptions for causal impact approach

There is a set of assumptions which sustain the validity of Causal Impact approach based on Bayesian Structural Time Series [40]. The assumptions can be postulated as follows.

i) State-space model: the model assumes a state-space framework where the observed time series is driven by underlying latent states that evolve over time according to a state transition equation.
ii) Linear Gaussian process: the evolution of latent states and the observation process are assumed to follow a linear Gaussian process, allowing tractable inference using Bayesian methods.
iii) Prior distributions: priors are assigned to model parameters, reflecting prior beliefs about their values. These priors are typically non-informative or weakly informative to let the data influence the posterior distributions significantly. Because the Causal Impact approach uses spike-and-slab, Bayesian model averaging and Markov Chain Monte Carlo to calculate parameter values. By default, uninformative priors are applied, meaning that all predictors are treated with equal likelihood of inclusion.
iv) No structural breaks in pre-intervention period: the model assumes no structural breaks or regime changes in the pre-intervention period, thus ensuring that the time series dynamics are stable and consistent before the intervention.
v) Sufficiently long pre-intervention period: the model requires a sufficiently long pre-intervention period to accurately estimate the underlying time series dynamics and predict the counterfactual scenario.

### Results interpretation and graphical representation of Causal impact

From an intuitive perspective, the analysis and interpretation of the Causal Impact approach are grounded in Bayesian statistics. The prior distribution represents the initial belief about the likelihood of specific outcomes and is informed by data collected prior to the intervention. The posterior distribution updates these prior beliefs based on new evidence observed after the intervention, providing a dynamic framework for understanding and predicting events. The estimation of the counterfactual outcome—the outcome that would have occurred in the absence of the intervention—is central to the analysis. This counterfactual is constructed by modelling the pre-intervention data and extrapolating it into the post-intervention period. The causal impact is quantified as the difference between the predicted counterfactual outcomes and the actual observed outcomes following the intervention. This methodology enables a rigorous assessment of the intervention’s effect while accounting for uncertainty inherent in the data.

The results are to be interpreted as a comparison between the observed and the counterfactual values, which are predicted according to the Bayesian structural time series model. The counterfactual represents the scenario which would have occurred without the implementation of the EAP in 2011 in Portugal. The results are presented in a format that allows comparison between observed and counterfactual estimated values for 2019, and for the post-treatment effect, both the average and cumulative response effects.

The main results also include the estimation of the Bayesian Credible intervals (CIs) and the one-sided tail-area probability which expresses the probability of obtaining this effect by chance (also referred as Chance probability). Additionally, the posterior probability of a causal effect represents the probability that the EAP intervention effect is indeed due to this intervention, as opposed to a random variation, in other words it quantifies the confidence in the causal attribution of the observed effect.

From an intuitive perspective, the Posterior probability quantifies the degree of uncertainty about causal effects. A high Posterior probability indicates strong evidence that the observed effect is attributable to the intervention rather than to random variation. This measure provides a probabilistic assessment of the true effect while also incorporating the uncertainty associated with that likelihood. The Chance probability, also known as the Bayesian one-sided tail-area probability, represents the probability that an observed effect could occur due to random chance rather than a real underlying cause. Conceptually, it answers the question: If there were no true effect, what is the likelihood of observing an effect as extreme as this or more? This metric reflects the uncertainty inherent in the counterfactual estimation. While the Chance probability evaluates the extremity of the observed data under the null hypothesis (that the intervention had no impact), the posterior probability directly assesses the likelihood of hypotheses after incorporating observed data and prior beliefs. Together, these indicators serve complementary roles in Bayesian inference, enhancing the interpretation of results by balancing assessments of data extremity and evidence strength within a contextual framework.

The results from Causal impact analysis are usually visually presented using three related graphs labelled Original, Pointwise, and Cumulative. The first graph, Original, represents observed values by a solid line, the counterfactual values by a dashed line, and a shaded area around counterfactual values representing the potential statistical variance (smaller areas represent more accurate prediction). The year of the implemented treatment, 2011, is represented by a vertical dashed line. The second graph is the Pointwise graph which shows the positive and negative causal impacts by calculating the difference between the counterfactual and the observed values. Finally, the third graph shows the Cumulative impact, that is, it displays the successive causal impacts since the implementation of the treatment. For the sake of space, these graphs are presented in the Appendix.

## Results

### Descriptive results

Table 3 presents the values for health outcome variables in 1999, 2011, and 2019 in Portugal. The observed values seem to show that the implementation of the economic and financial assistance programme in 2011 did not impact population health negatively, in general, as health indicators continue to show an improving trend over time, as does the percentage change between 2011–2019.

**Table 3:** Descriptive statistics for health indicators, Portugal.

	1990	2011	2019	Percentage change (1990–2011)	Percentage change (2011–2019)	Percentage change (1990–2019)
**DALY**	28 219.31	21 184.31	19 673.9	-24.93	-7.13	-30.28
**HLY**	65.69	69.8	70.96	6.26	1.66	8.02
**LEB**	74.4	79.9	81.1	7.39	1.50	9.01
**LE65**	16.3	19.9	20.6	22.09	3.52	26.38
**IMR**	9.2	3.1	2.8	-66.30	-9.68	-69.57
**CVD_D**	292.48	111.3	95.01	-61.95	-14.64	-67.52
**Respiratory_D**	28.86	29.84	27.51	3.40	-7.81	-4.68
**Stroke_D**	193.08	65.39	55.97	-66.13	-14.41	-71.01
**Cancer_D**	126.64	118.85	114.54	-6.15	-3.63	-9.55
**HIV_D**	3.43	4.38	2.23	27.70	-49.09	-34.99
**TB_D**	3.83	1.81	0.89	-52.74	-50.83	-76.76

Nevertheless, it can also be seen that the rate of improvement in health outcomes slowed down from 2011 on. For some indicators, the improvement is remarkable in the period 1990–2011, but it is much less significant after 2011, as observed for infant mortality rate, CVD, and stroke mortality. The health indicator for lower respiratory infection deaths, which largely accounts for pneumonia mortality, showed a rather different pattern as it registered some deterioration before 2011 and an improvement after this year. For the infectious diseases’ mortality, HIV/AIDs and tuberculosis deaths, there was an improvement for the whole period 1990–2019. However, while deaths from tuberculosis saw a decrease before and after 2011, deaths from HIV/AIDS only registered a decrease after 2011.

**Graph 2:**
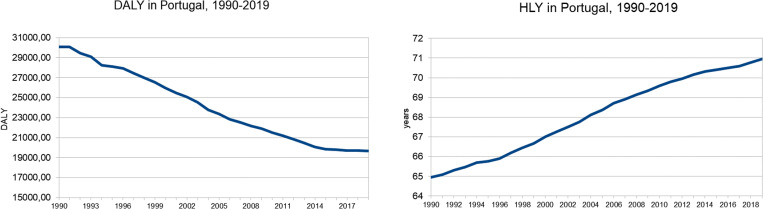
DALY and HLY in Portugal, 1990–2019. The first graph shows a decreasing trend of DALY and the second graph shows an increasing trend of HLY.

It should be noted that the evolution of DALY and HLY is nearly symmetrical over time period 1990–2019, as expected. While the trend for DALY is decreasing, the trend for HLY is increasing, as shown in Graph 2. These two health indicators are very relevant general measures for population health and provide very good indication on how the population health is evolving [53].

In sum, it is observable that after 2011 there is a general long-run trend for the improvement of health indicators. The question is to know if there is any impact on this trend after 2011, resulting in a slowdown of these population health improvements.

To end the descriptive results analysis, Table 4 presents the values for control variables in 1999, 2011, and 2019 in Portugal. Between 1990 and 2019, Portugal experienced marked demographic and educational shifts, evidenced by a substantial rise in the proportion of individuals aged 65 and older and a fourfold increase in the share of the population holding a university degree. Over the same period, CO₂ emissions exhibited minor fluctuations but followed an overall downward trajectory.

**Table 4:** Control variables descriptive values, Portugal.

	1990	2011	2019
% population +65	13.2	18.7	21.8
% population tertiary level of education	3.36	13.4	20.0
CO2 emissions	5.44	5.2	4.9

### Causal impact results

Results obtained from causal analysis show that in general the implementation of the structural adjustment programme in 2011 resulted in a negative impact on population health, meaning a deceleration of health improvements. The key general health outcome indicators reveal that the counterfactual estimates tend to diverge from observed ones in an unfavourable way, suggesting that, in the absence of the implementation of the structural adjustment programme, health outcome indicators would likely to have shown more favourable trajectories.

A general overview of the results is presented in Table 5. This table shows the qualitative assessment of the causal impact on the health indicator where ‘negative’ means that there is a causal impact which either worsens health or decelerates health improvements; the original and counterfactual values in 2019; the average response and cumulative response for the period after the EAP implementation (2012–2019); the difference between the average observed and expected response in both cases (average response and cumulative response); and finally, the Chance probability. Additionally, the posterior probability of a causal effect is also provided in the last column of Table 5.

**Table 5:** Summary of main causal effects.

			During the period after bailout programme			
	Estimated Causal Impact^a^	Observed and counterfactual values in 2019	Average response 2012-2019	Sum of values (cumulative) 2012-2019	Chance probability^b^ (%)	Posterior probability (%)
General health indicators						
DALY	Negative	Observed:19 673.92 Estimated:18 166.44	Observed: 20.01K Estimated: 19.09K (95% CI: [15.61K,21.81K]) Effect size: 0.92K (95% CI: [-1.81,4.40K])	Observed: 160.06K Estimated: 152.72K Effect percentage: + 5%	27	75
HLY	Negative	Observed:70.96 Estimated:71.87	Observed: 70.46 Estimated: 71.06 (95% CI: [69.66,72.85]) Effect size: -0.60 (95% CI: [-2.39,0.80])	Observed: 563.69 Estimated: 568.51 Effect percentage: -1%	21	76
LEB	Negative	Observed:81.1 Estimated:82.2	Observed: 80.54 Estimated: 81.11 (95% CI: [79.40,83.51]) Effect size: -0.57 (95% CI: [-2.97,1.14])	Observed: 644.30 Estimated: 648.84 Effect percentage: -1%	29	71
LE65	Negative	Observed:20.6 Estimated:21.33	Observed: 20.14 Estimated: 20.50 (95% CI: [19.49,22.28]) Effect size: -0.36 (95% CI: [-2.14,0.65])	Observed: 161.10 Estimated: 164.00 Effect percentage: -2%	38	63
Specific mortality indicators						
IMR	Negative	Observed:2.8 Estimated:0.12	Observed: 3.01 Estimated: 1.36 (95% CI: [-2.15,3.88]) Effect size: 1.65 (95% CI: [-0.87,5.17])	Observed: 24.10 Estimated: 10.90 Effect percentage: + 121%	24.5	76
CVD_D	Negative	Observed:95.01 Estimated:64.81	Observed: 103.58 Estimated: 84.93 (95% CI: [19.36,139.72]) Effect size: 18.66 (95% CI: [-36.14,84.23])	Observed: 828.67 Estimated: 679.43 Effect percentage: + 22%	23	76
Respiratory_D	Positive	Observed:27.51 Estimated:33.01	Observed: 28.77 Estimated: 31.72 (95% CI: [28.30,37.74]) Effect size: -2.95 (95% CI: [-8.97,0.47])	Observed: 230.17 Estimated: 253.75 Effect percentage: -9%	4.5	96
Stroke_D	Negative	Observed:55.97 Estimated:10.26	Observed: 56.77 Estimated: 28.61 (95% CI: [-22.03,72.78]) Effect size: 28.16 (95% CI: [-16.01,78.80])	Observed: 454.18 Estimated: 228.89 Effect percentage: + 98%	9	92
Cancer_D	Negative	Observed:114.54 Estimated:111.67	Observed: 115.67 Estimated: 113.62 (95% CI: [110.52,116.09]) Effect size: 2.05 (95% CI: [-0.42,5.15])	Observed: 925.35 Estimated: 908.97 Effect percentage: + 2%	6	93
HIV_D	Negative	Observed:2.23 Estimated:0.66	Observed: 2.72 Estimated: 1.72 (95% CI: [-1.34,5.54]) Effect size: 1.00 (95% CI: [-2.82,4.06])	Observed: 21.76 Estimated: 13.77 Effect percentage: + 58%	24	77
TB_D	Negative	Observed:0.89 Estimated:0.53	Observed: 0.98 Estimated: 0.75 (95% CI: [-0.35,1.35]) Effect size: 0.24 (95% CI: [-0.37,1.33])	Observed: 7.88 Estimated: 5.98 Effect percentage: + 32%	26	75

Note: a) Qualitative assessment of the difference between estimated counterfactual and original values; b) ‘Chance probability’ is the Bayesian one-sided tail-area probability which expresses the probability of obtaining this effect by chance; c) CI is Credible interval.

The analysis of DALY and HLY indicates that, following 2011, counterfactual estimates generally reflect more favourable health outcomes than those observed (Table 5). This pattern, indicative of a negative impact on population health, is consistent across most health indicators, with the exception of deaths from lower respiratory infections, which exhibit an inverse causal relationship (Table 5).

For DALY indicator, the observed average value was 20,010, while the counterfactual mean was estimated at 19,090, with a 95% credible interval of (15,610; 21,810). The resulting effect size is equal to 920, corresponding to a 5% higher accumulated DALY burden. Regarding HLY, the observed average was 70.46 years compared to a counterfactual average of 71.06 years within a 95% credible interval of (69.66; 72.85); the absolute effect size is −0.60 years, representing a 1% relative decline in HLY after the implementation of the EAP.

Posterior tail-area probabilities, or chance probabilities, for DALY (26%) and HLY (21%) suggest that a small portion of the posterior distribution lies within the extreme tails, implying that random variation may partially explain the observed effects. Nevertheless, the posterior probabilities of 75% for DALY and 76% for HLY support the hypothesis that the intervention exerted a causal.

These findings suggest a moderate-to-high probability that the EAP affected health outcomes, despite the presence of some uncertainty. The posterior probabilities, derived from the combination of prior information and observed data, underscore the role of both elements in Bayesian inference; thus, although the observed data alone may not provide strong statistical support for the hypothesis, the incorporation of prior knowledge yields a moderate-strong level of confidence in its validity.

**Graph 3:**
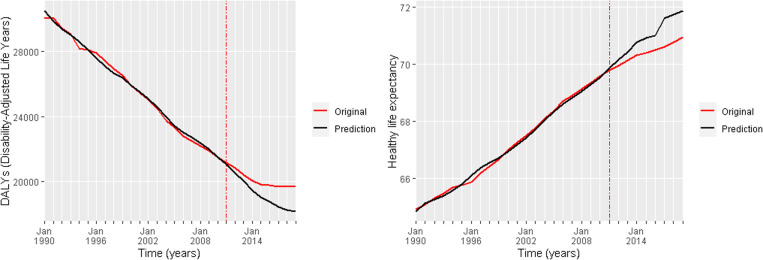
Observed and counterfactual DALY and HLY values. The first graph refers to DALY and the second graph refers to HLY values both original and predicted.

Still focusing on health indicators DALY and HLY, Graph 3 shows the visual comparison between the observed (or original) and the counterfactual values. These two graphs clearly show that had there not been an EAP, population health would be at better levels.

A detailed view of all findings obtained are shown in Graphs A1-A4, in the Appendix, for all the health indicators. These graphs show the Original, Pointwise, and Cumulative series for each variable: DALY, HLY, LEB, LE65, IMR, CVC_D, Respiratory_D, Stroke_D, Cancer_D, HIV_D, and TB_D.

## Discussion

During the European debt crisis in 2011 Portugal was subject to an Economic and Adjustment Programme (EAP) which aimed to reduce the weight of public spending in the economy. The health sector was one of the most affected sectors for which the aim was to generate expenditure savings and create mechanisms for the future control of health expenditure. The negative short-run effects on health accruing from the implementation of this programme are explained in some studies. However, the causal long-run effects on health of this programme are barely known and this study aimed to assess this causal impact.

The evidence obtained by estimating a counterfactual series of health outcome indicators, which express the probable scenario without the implementation of the EAP, shows that in general the causal impact was negative, meaning that, without the implementation of the EAP, health indicators would have been better, despite some level of uncertainty associated with this impact. In some instances, the contextual analysis may be needed to understand and interpret the obtained findings.

The transmission pathways [18] of this negative causal impact emerged from: i) the direct negative impact on the healthcare system, which become not only underfunded but also provided less and lower quality health care [9,11]; ii) the indirect negative impact related to other policies, such as those linked to the labour market, education, employment, and the housing market [5,13]; and finally, iii) the multiplicative negative direct effect of social and economic determinants of health, such as unemployment and poverty [6,7,10]. Some of these effects may be observable in the short-run, but they become clearer and magnified in the long-run, as effects accumulate over a long time.

This study is unique in this strand of the literature, not only because it explores the long-run causal effects of implementing the EAP, but also because it uses a Bayesian structural time-series. Previous studies for Portugal have shown the short-run associations resulting during and right after the implementation of the EAP. These studies already pointed to a negative effect on health measured by higher mortality rates from respiratory diseases, suicide and road traffic, or by incidence rates of infectious diseases [10], although no causality was established. Evidence obtained in the current analysis shows that, in fact, some of those correlations tend to reflect a causality pattern over time. But not all of them. No causal impact was found for lower respiratory infection deaths, which is not aligned with the previous proposal [10] that suggested an increase mortality rate due to respiratory diseases. One possible explanation for this later finding could arose from the existence of other public intervention which produced a stronger and opposite effect.

Concerning lower respiratory infection deaths, which include a large share of pneumonia mortality, the Portuguese background for this disease needs clarification. Portugal has one of the worst mortality rates for pneumonia across European countries [54], which has attracted the attention of population health authorities. Moreover, respiratory diseases are the third or fourth most important cause of mortality in the country, depending on the period considered [55–57]. So, since 2012 there has been a national programme of respiratory diseases aimed to reduce the mortality figures. It may be that the effect of this programme overcomes the negative effect from EAP.

Regarding the causal impact found in the other health outcomes, some important comments are relevant. First, for two notable health outcome indicators, DALY and HLY, there is strong evidence of deterioration caused by the EAP. These two indicators are two sides of the same coin, providing a robustness check for this finding: in the absence of the EAP the level of DALY would have been lower while the values for HLY would have been higher.

Second, by the same token, results show a slower improvement of life expectancy which is partially explained by the implementation of the bailout programme. The evidence shows a clearer impact on the life expectancy at birth than on the life expectancy at 65. Additional and complementary findings reveal a differential impact by gender (see Graphs A7 in the Appendix). The effect of the EAP is more pronounced for women, suggesting that, in its absence, women would have experienced a faster increase in life expectancy at age 65 (LE65) compared to men. This result aligns with existing evidence indicating that women are disproportionately affected by economic crises [58].

The third comment refers to specific causes of death for which the counterfactual estimates show better values: infant rate of mortality, CVD, stroke, and cancer deaths. Infant mortality rate data, in Portugal, shows some stabilizing level in the period after 2011, about 3 deaths per 1000 births, which could be considered as the minimum possible achievable given the technological and medical conditions [42]. In fact, the lowest infant mortality rate in the world is around 1.5–2.0 deaths per 1000 births [42,55]. Despite displaying one of the best values for infant mortality rate across the world, there is some room for improvement in Portugal. Therefore, the counterfactual values are only partially acceptable under the current social, economic and medical conditions as the observable values are already at an excellency level.

Cardiovascular diseases (CVD), stroke and cancer are top causes of mortality in Portugal [53–56]. These non-communicable diseases share some risk social factors such as low income, unemployment, poverty, and poor housing conditions [59] which were harshly hit with the EAP [5–7,13]. Additionally, the budget cuts in the health sector which resulted in longer waiting lists, higher levels of unmet health care needs, and lower number of cancer screenings [10,53] also contributed to the deterioration of people health, which over time became clearer as symptoms appeared and intensified, and diseases emerged. The causal analysis for CVD, stroke, and cancer deaths shows without the implementation of the EAP these rates would have progressed towards lower levels of mortality rate.

Finally, concerning infectious disease indicators for HIV/AIDS and tuberculosis, the findings show that EAP has caused a worse evolution for both causes of mortality. Portugal has one of the highest figures for the mortality caused by HIV/AIDS and tuberculosis among European countries [53–57,60]. In 2019 the country ranked fourth and third among EU countries for the highest mortality rates due to tuberculosis and HIV/AIDS, respectively [54,57,60]. Despite the national efforts invested in national programmes to improve these numbers, the results have been weak, and the two death rates are correlated due to co-infection [60,61]. Determinant factors for the incidence and prevalence of these diseases were exacerbated during the EAP period, and included unemployment, poverty, limited access to health care. These disadvantage determinants have certainly contributed to lowering the effectiveness of the national programmes aimed to improve health outcomes related to tuberculosis and HIV/AIDS.

### Limitations and strengths

The findings obtained in this study suffer from a high level of uncertainty derived from both moderate-high values of the Posterior probability and Chance probability [31] which calls for an assessment within a contextual framework.

Firstly, concerning data, the series include 30 years of observed data and only 8 years of post-treatment period. So it could be that 8 years is too short a time to detect a long-term health effects resulting from the EAP. However, on the one hand, 8 years is long enough for some diseases to cause burden as cancer and cardiovascular disease. On the other hand, a longer post-treatment period cannot be established due to the COVID-19 pandemic.

Second, data may show some variability, as happens with cancer, lower respiratory infectious disease, and tuberculosis deaths, which creates noise that makes it difficult for the algorithm to distinguish between random fluctuations and actual intervention effects.

Third, it could be that the control variables do not effectively capture the factors influencing the outcome. However, it is well-accepted that education, demographic structure, and pollution influence health outcomes. But maybe within this period these determinants were not the most influential determinants, despite being the only ones that were not directly affected by the implementation of the EAP.

Fourth, the estimated model assumes uninformative priors, ensuring that counterfactual estimates are driven primarily by the observed data rather than by subjective prior assumptions. Modifying these priors to be more informative, such as by reducing variance or adjusting inclusion probabilities, would yield different posterior distributions. However, the use of uninformative priors allows the posterior to reflect the observed data more directly, which is particularly appropriate for limited datasets, even if it introduces greater uncertainty.

Finally, it is assumed that the relationship between covariates and treated time series established during the pre-treatment period remains stable throughout the post-period. But given the troubled period after the implementation of the EAP, it might be that this assumption was not verified, but there are no straightforward means to verify it.

Despite its limitations, this study provides the first causal evidence of the impact of the 2011 Economic and Adjustment Programme (EAP) in Europe. Its findings are unique and complement prior research that has reported associations and correlations related to EAP implementation in European countries [5,6,8–12], as well as cross-country comparisons based on instrumental variable approaches in contexts involving IMF financial support [14,15].

## Conclusion

The evidence indicates a negative long-term causal impact of the 2011 Economic Adjustment Programme on population health in Portugal. These findings support the need for integrating social and health policies alongside IMF programmes or other forms of financial assistance to indebted countries, in order to mitigate adverse long-term effects on health.
